# NeuroSAFE-guided robot-assisted radical prostatectomy versus standard RARP: systematic review and meta-analysis of comparative studies

**DOI:** 10.1007/s11701-026-03390-w

**Published:** 2026-05-13

**Authors:** Caio Vinicius Suartz, José Pedro Cassemiro Micheleto, Bianca Espadilha Condotta, Leonam Bringhenti Schumacher, Ketlyn Assunção Galhardo, Maria Fernanda Dias Azevedo, Pedro Henrique Souza Brito, Daher Chade, José Maurício Mota, Maurício Cordeiro Dener, Thalita Bento Talizin, Walid Sharour, Walid Shabana, Roberto Iglesias Lopes, Marian Wettstein, Mohamed Elkoushy, William Carlos Nahas, Bárbara Vieira Lima Aguiar Melão, Murilo de Almeida Luz, Leonardo Oliveira Reis, Jose de Bessa Junior, Roni De Carvalho Fernandes, Leopoldo Alves Ribeiro Filho

**Affiliations:** 1 Department of Urology, Northern Ontario School of Medicine University, Thunder Bay, ON Canada; 2 Division of Urology, Institute of Cancer of São Paulo, University of São Paulo, São Paulo, Brazil; 3 Genitourinary Medical Oncology Service, São Paulo State Cancer Institute, University of São Paulo, São Paulo, Brazil; 4 Instituto D’Or de Pesquisa e Ensino, São Paulo, Brazil; 5 Departamento de Oncologia, Hospital Adventista de Manaus, Manaus, Amazonas Brasil; 6 Department of Urology, Icahn School of Medicine at Mount Sinai, New York, NY USA; 7 UroScience, School of Medical Sciences, University of Campinas (UNICAMP), Campinas, São Paulo, Brazil; 8 ImmunOncology, Pontifical Catholic University of Campinas (PUC-Campinas), Campinas, São Paulo, Brazil; 9 INCT UroGen, Technology and Innovation in Genitourinary 20 Cancer, National Institute of Science, Campinas, São Paulo, Brazil; 10 Department of Surgery, State University of Feira de Santana, Feira de Santana, Brazil; 11 School of Medical Sciences, Santa Casa de São Paulo, São Paulo, Brazil

**Keywords:** Prostate cancer, Robot-assisted radical prostatectomy, NeuroSAFE, Meta-analysis, Prostatectomy

## Abstract

Prostate cancer is the most common male malignancy, and although robotic-assisted radical prostatectomy (RARP) is widely used, urinary incontinence and erectile dysfunction remain significant issues. NeuroSAFE, an intraoperative frozen section technique aimed at optimizing nerve preservation without compromising cancer control, is increasingly used, but its overall impact on outcomes remains unclear.To systematically evaluate the effectiveness and safety of the NeuroSAFE technique during RARP, comparing functional recovery and oncologic outcomes with non-NeuroSAFE approaches.This systematic review and meta-analysis followed PRISMA 2020 guidelines and was registered in PROSPERO (CRD420251032774). A comprehensive search of Embase, MEDLINE, and ClinicalTrials.gov (March, 2026) identified studies reporting oncologic and/or functional outcomes in men undergoing RARP with NeuroSAFE. Two reviewer teams independently conducted study selection, data extraction, and risk-of-bias assessment using ROBINS-I tool. Random-effects meta-analyses estimated pooled odds ratios (ORs) with 95% confidence intervals (CIs). Heterogeneity was quantified with the I^2^ statistic, and publication bias was evaluated using Egger’s test.Thirteen studies (2 randomized controlled trials and 11 observational cohorts) comprising 22,183 patients met the inclusion criteria. NeuroSAFE was associated with significantly improved postoperative erectile function (OR 2.00; 95% CI 1.46–2.74) and urinary continence recovery (OR 1.36; 95% CI 1.05–1.76). Positive surgical margins were significantly reduced in the NeuroSAFE group (OR 0.73; 95% CI 0.59–0.89). No differences in biochemical recurrence were observed (OR 0.81; 95% CI 0.43–1.56). Heterogeneity was substantial for some outcomes, and most nonrandomized studies carried a serious risk of bias, which limits causal inference.This systematic review and meta-analysis, including randomized evidence, demonstrates that NeuroSAFE is a safe and effective intraoperative strategy that is associated with improved erectile function, enhanced continence recovery, and negative surgical margins without compromising oncologic outcomes. These findings support integrating NeuroSAFE into surgical decision-making for patients undergoing RARP, particularly when nerve preservation can be achieved without compromising oncologic outcomes.In this study, we compared a surgical approach that uses real-time analysis during prostate cancer surgery with the standard technique. This method helps surgeons decide how much tissue to preserve while the operation is still ongoing. We found that this approach is safe and may improve recovery of urinary continence and sexual function, while also reducing the risk of leaving cancer behind, without compromising cancer control.

**Supplementary Information:**

The online version contains supplementary material available at 10.1007/s11701-026-03390-w.

## Introduction

Prostate cancer (PCa) is the most frequently diagnosed malignancy among men worldwide, accounting for approximately 30% of all cancer diagnoses [1]. For localized disease, robotic-assisted radical prostatectomy (RARP) has become the predominant surgical approach, largely replacing open radical retropubic prostatectomy (RRP) [2]. Despite its technical advancements, postoperative urinary incontinence and erectile dysfunction remain the most common adverse outcomes following radical prostatectomy. In a phase 3 randomized trial, Yaxley et al. found no significant difference in domain-specific quality of life or pathological outcomes at 12 weeks between RARP and RRP when both procedures were standardized for all relevant parameters [3]. 

While age and baseline erectile status are the primary determinants of postoperative sexual recovery, nerve-sparing (NS) has additionally been correlated with enhanced continence outcomes and may retain value in patients with limited preoperative erectile function [4, 5].

The foundation of oncologic surveillance after radical prostatectomy (RP) is serial measurement of prostate-specific antigen (PSA), as biochemical relapse typically precedes any clinical evidence of recurrence. Patients who experience biochemical recurrence tend to present with more adverse baseline and pathological features, including higher median PSA at diagnosis, a greater prevalence of Gleason grade ≥3+4, increased rates of extraprostatic extension, and a higher frequency of positive surgical margins (PSMs) compared with those who remain recurrence-free [6]. 

To improve functional outcomes without compromising oncologic control, surgical strategies that focus on preserving the neurovascular bundles (NVBs), which contain parasympathetic fibers from the pelvic plexus, have been developed. Among these, the NeuroSAFE technique, introduced by [26], is a standardized intraoperative frozen section (IFS) protocol that enables real-time assessment of the prostate resection margin. This approach facilitates maximal nerve preservation while minimizing the risk of PSMs [7].

We conducted a systematic review with meta-analysis of studies evaluating the NeuroSAFE approach during RARP and its association with oncologic and functional results.

## Methods

### Protocol and registration

The systematic review and meta-analysis was performed in line with the PRISMA 2020 guidelines [8] and it was prospectively registered in PROSPERO (CRD420251032774; April 28, 2025) [9].

A comprehensive search of the Embase, MEDLINE, and ClinicalTrials databases was performed on March 13, 2026. Detailed search strategies are provided in the Supplementary Material.

### Eligibility criteria

Studies were included if they enrolled men who underwent RARP for PCa using the NeuroSAFE technique and compared with RARP patients without the NeuroSAFE technique, and reported oncologic and/or functional outcomes. Definitions of functional and oncological outcomes and assessment criteria are detailed in Supplementary Table 1. Case reports describing technical details, systematic and narrative reviews, conference proceedings, editorial letters, and abstracts were excluded. No language restrictions were applied.

### Selection process

Two reviewers (KG and PB) independently screened all records for eligibility. Disagreements were resolved through discussion with a third author (CS) until consensus was achieved. When overlapping data or duplicate publications were identified, only the study with the largest cohort, longest follow-up, or most recent dataset was included.

### Data collection process

Two reviewers (KG and PB) independently performed study selection in three sequential phases: title screening, abstract evaluation, and full-text review. Reference lists of included articles were also searched to capture additional eligible studies through citation chaining. Any disagreements were resolved by consensus with a third author (CS). All screening and coding procedures were conducted using Rayyan software [10].

### Data items

Data were independently extracted into a pretested spreadsheet (Google Sheets 2025; Google LLC). The extracted variables included: first author, article title, journal, publication year, study design, surgical approach, sample size, study objectives, nerve-sparing laterality, surgery duration, and parameters related to oncologic and functional outcomes.

### Effect measures

A meta-analysis for the functional and oncological outcomes was conducted using R version 4.3.2 (R Foundation for Statistical Computing, Vienna, Austria) [11], primarily with the meta package for pooled analyses and graphical outputs. The dmetar package was used to perform Egger’s test for publication bias, and readxl was used for data import.

For dichotomous outcomes, we calculated odds ratios (ORs) with corresponding 95% confidence intervals (CIs), using unadjusted estimates or, when available, adjusted values from multivariable models. A two-sided p value <0.05 was considered indicative of statistical significance.

### Synthesis methods

Statistical heterogeneity was assessed using Cochran’s Q test and the I^2^ statistic [12]. I^2^ values were categorized as 0% to <25% (negligible), 25% to <40% (moderate), and ≥40% (substantial heterogeneity). A random-effects model was applied when I^2^ was ≥40%, in accordance with established methodological guidance [13]. A p-value of <0.10 was considered statistically significant for Cochran’s Q test [12].

### Study methodological quality assessment

**Fig. 1:**
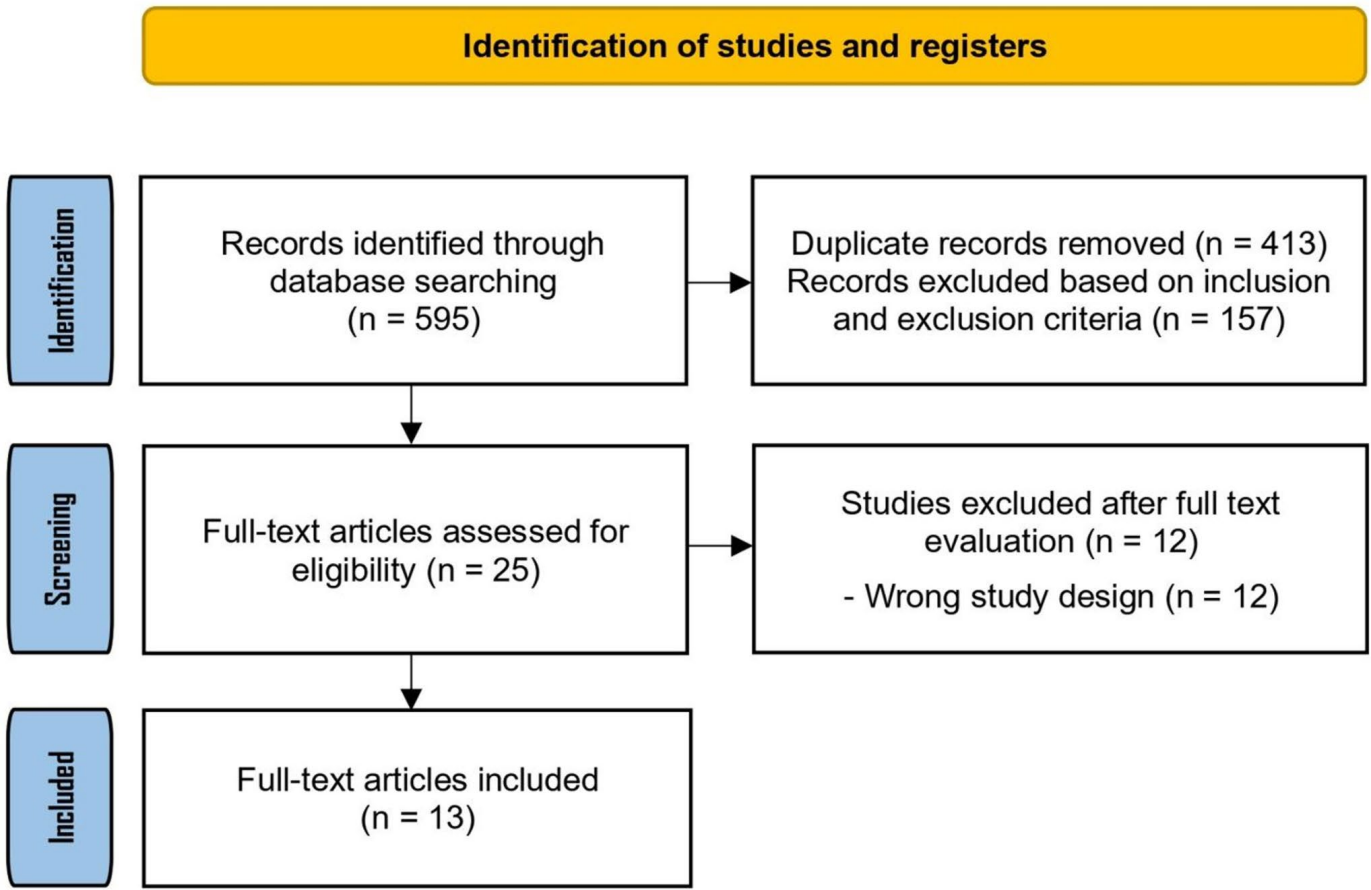
PRISMA

Two reviewers independently evaluated the risk of bias for each included study. Any discrepancies were resolved through discussion and, when necessary, with input from a third author. The *Risk of Bias in Non-randomized Studies of Interventions* (ROBINS-I) tool was applied, following the recommendations of the *Cochrane Handbook for Systematic Reviews of Interventions* for observational designs [14, 15]. Two randomized controlled trials were included [16, 17]; therefore, the RoB 2 tool was applied to assess the risk of bias in the randomized studies (Fig. 1).

**Fig. 2:**
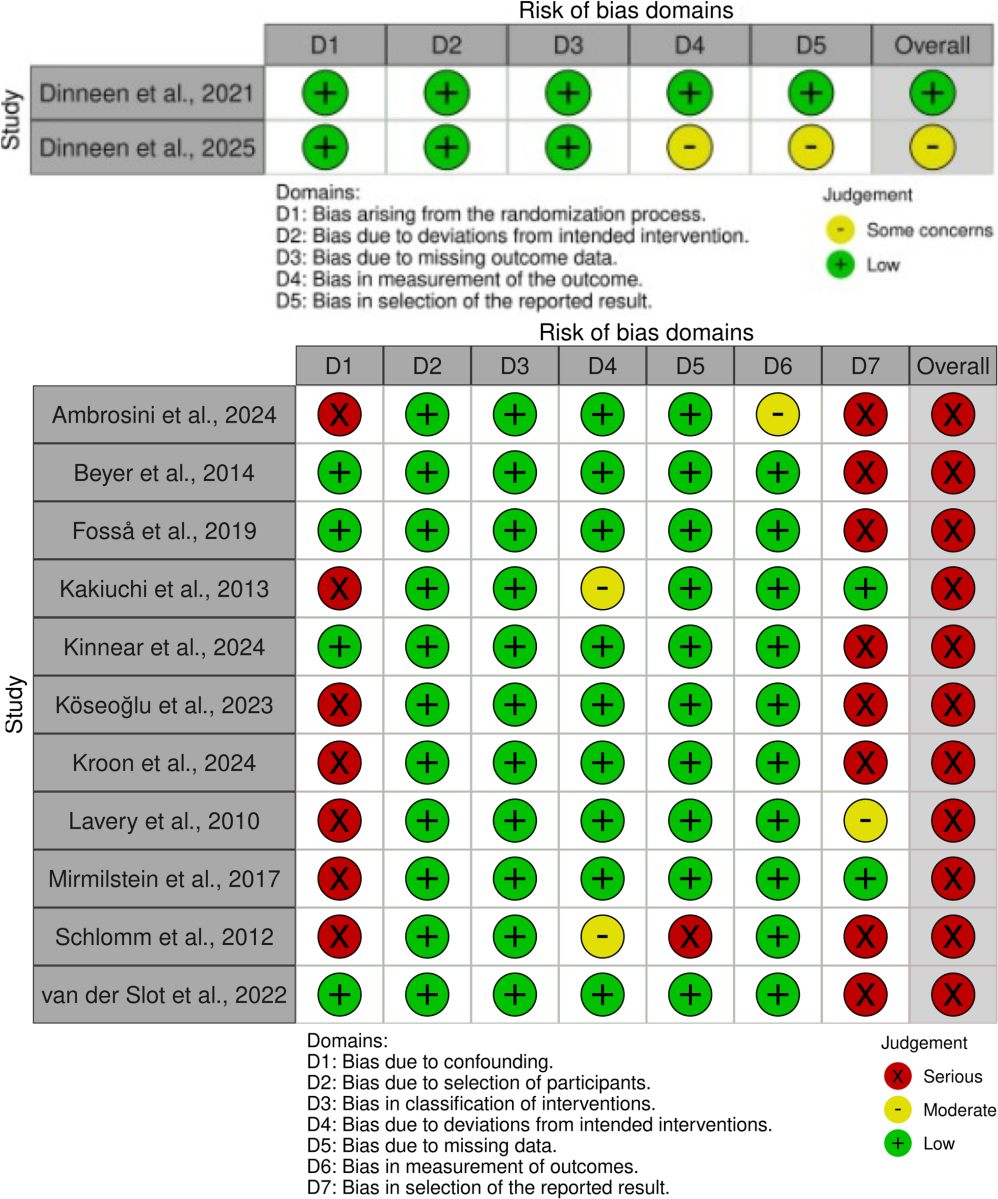
BIAS analysis

Figure 2 summarizes the methodological quality of the 13 included studies. The randomized trial was rated between low and *some concerns* for risk of bias [16, 17], whereas all nonrandomized cohort studies showed an overall *serious* risk of bias [18–28].

Blinding of patients or surgeons was not performed in any of the nonrandomized studies. Because surgeons must wait for IFS feedback, blinding is inherently impractical in the NeuroSAFE protocol. Consequently, this limitation may lead to measurement bias, especially in subjective endpoints such as continence and erectile recovery. 

## Results

### Study selection

The database search retrieved 595 records. We analyzed 25 full-text articles, and 13 studies met the inclusion criteria and were incorporated into the final analysis [16–28]. The study selection process is detailed in the PRISMA flow diagram (Fig. 1). Among the included studies, two were randomized controlled trials [16, 17], while the remaining were observational—eight retrospective [18, 20, 22–24, 26–28] and three prospective [19, 21, 25]. An overlap between the cohorts reported by Ambrosini et al. [28] and Schlomm et al. [26] was identified. 

### Study characteristics

Overall, thirteen studies published between 2012 and 2025 were included, encompassing a total of 22,183 patients. These studies were conducted across six countries: the United States, Germany, the United Kingdom, Norway, the Netherlands, and Turkey. Baseline characteristics, including patient age and nerve-sparing status, are summarized in Table 1 and Supplementary Material. Oncologic and functional parameters, such as preoperative PSA, Gleason score, erectile function, continence, PSMs, and biochemical recurrence, are presented in Table 2.

**Table 1:** Study characteristics

Study (N = 13)	Country	Study period	Type of study	NS			No. patients	Age (yrs)	
				Unilateral N (%)	Bilateral N (%)	Non-NS N (%)		NS	Non-NS
[24]	USA	May/2007 - September/2009	Retrospective cohort	63 (7)	853 (88)	46 (5)	970	58.6	59.3
[7]	Germany	January/2002 - June/2011	Retrospective cohort	-	-	-	11 069	-	
[20]	USA	June/2004 - September/2011	Retrospective cohort	-	-	-	2608	59.48 (SD: 7.23)	61.61 (SD: 6.84)
[18]	Germany	2004 - 2013	Retrospective cohort	-	-	-	1570	-	-
[25]	UK	November 2008 - February 2017	Prospective observational comparative study	87 (31,4)	187 (67,5)	-	277	58	62
[19]	Germany and Norway	2013-2016	Prospective cohort	242 (59%)	165 (40)	-	407	61 (IQR: 47–75)	64 (IQR: 44–75)
[17]	UK	May/2018 - March/2019	Prospective randomized controlled feasibility study	-	-	-	49	57	55.9
[16]	UK	January/2019 - December/2022	Randomized controlled trial	173 (45,5)	121 (31,8)	86 (22,6)	381	57.8 (SD: 6.4)	57.3 (SD: 7.0)
[27]	Netherlands	January/2016 - December/2020	Retrospective control and Neurosafe prospective	501 (28%)	894 (50)	361 (20)	1756	68 (IQR: 63–71)	66 (IQR: 61-70)
[22]	Turkey	2019	Retrospective cohort	50 (24,0)	141 (67,7)	20 (9,6)	208	63.4 (SD: 0.7)	63.7 (SD: 0.6)
[23]	Netherlands	September/2018 - January/2021	Retrospective cohort	-	-	-	1797	68 (IQR: 63-71)	64.6 (IQR: 60-68)
[21]	UK	01/2009 - 06/2018	Prospective cohort	-	-	-	1140	59,9	64,7
[28]	Germany	January 2002 – June 2011	Retrospective cohort	854 (33.3)	1626 (63.3)	-	3014	64 (59–68)	64 (59–68)

IQR = Interquartile Range; SD = Standard Deviation; NS = Nerve-sparing; UK = United Kingdom; USA = United States of America

**Table 2:** Oncologic characteristics of the included studies

Study (n = 13)	Pre-op PSA NeuroSAFE	ISUP NeuroSAFE N (%)/total	Erectile function outcome N (%)/Total	Continence outcome N (%)/total	Positive surgical margins N (%)/total	BCR positive N (%)/total
[24]	5.7	[1] = 50 (28)/177 [≥2] = 120 (68) / 177 [≥4] = 7(4)/177	786 (81)/970	892 (92)/970	Focal = 108 (11)/970 Extensive = 52 (5)/970 Total = 160 (16)/970	38 (5)/970
[7]	-	[1] = 751 (13.9)/5392 [2] = 3560 (66.0)/5392 [3] = 835 (15.4)/5.392 [≥4] = 411 (7.6)/5.392	-	-	947 (36.8)/2567	-
[20]	6.50 (5.46)	[1] = 634 (56,2%)/2608 [2/3] = 401 (35,5%)/2608 [≥4] = 86 (7,6%)/2608	-	-	272 (10,4%)/2608	-
[18]	-	[1] = 128 (10.9)/1178 [2] = 810 (68.8)/1178 [3] = 202 (17.2)/1178 [4] = 37 (3.1)/1178	-	-	180 (18.7)/961	-
[25]	7.37 (3.64)	[1] = 31 (25.8)/120 [2] = 62 (51.7)/120 [3] = 17 (14.2)/120 [≥4] = 10 (8.3)/120	86 (35,1)/245	182 (92,3)/197	39 (14,0)/277	5 (1.8)/277
[19]	-	-	175 (42,9)/407	175 (42,9)/407	64 (15,7)/407	
[17]	10.4 (Range 51-66)	[1] = 1 (4)/25 [2] = 19 (76)/25 [3] = 5 (20)/25 [4] = 0/25 [5] = 0/25	-	-	-	
[16]	-	[1]: 92 (9.6%)/959 [2]: 495 (51.6%)/959 [3]: 269 (28%)/959 [4]: 49 (5.1%)/959 [5]: 54 (5.6%)/959	-	-	510 (29,0)/1.756	199 (17.8%)/1112
[27]	6.7 (0.5)	[1] = 31 (34.4)/90 [2] = 33 (36.6)/90 [3] = 15 (16.6)/90 [4] = 7 (7.7)/90 [5] = 4 (4.7)/90		-	57 (27.4)/208	-
[22]	-	[1] = 87 (9)/962 [2] = 501 (52)/962 [3] = 271 (28)/962 [4] = 49 (5.1)/962 [5] = 54 (5.6)/962		-	559 (31,1)/1797	-
[23]	-	[1] = 107 (32,3)/331 [2] = 153 (46,2)/331 [3] = 34 (10,2)/331 [4] = 21 (6,3)/331 [5] = 2 (0,6)/331		828 (72%)/1140	193 (16,9)/1140	-
[21]	9.1 (6.5)	[1] = 9 (5)/189 [2] = 140 (74)/189 [3] = 26 (14)/189 [4] = 10 (5)/189 [5] = 4 (2)/189		-	120 (31,4)/381	-
[28]	-	-	182 (68.0)/276	294 (92.4)/318	10.6%/1507	-

BCR = Biochemical Recurrence; PSA = Prostate Specific Antigen; Pre-op = Preoperative

### Risk of Bias Assessment

Authors’ judgments about each domain for each included study are exposed in Fig. 2. The two randomized controlled trials were judged to have low to moderate risk of bias, whereas the eleven nonrandomized cohort studies demonstrated serious methodological limitations across several domains [16–28].

### Results of syntheses

#### Surgery duration

**Fig. 3:**
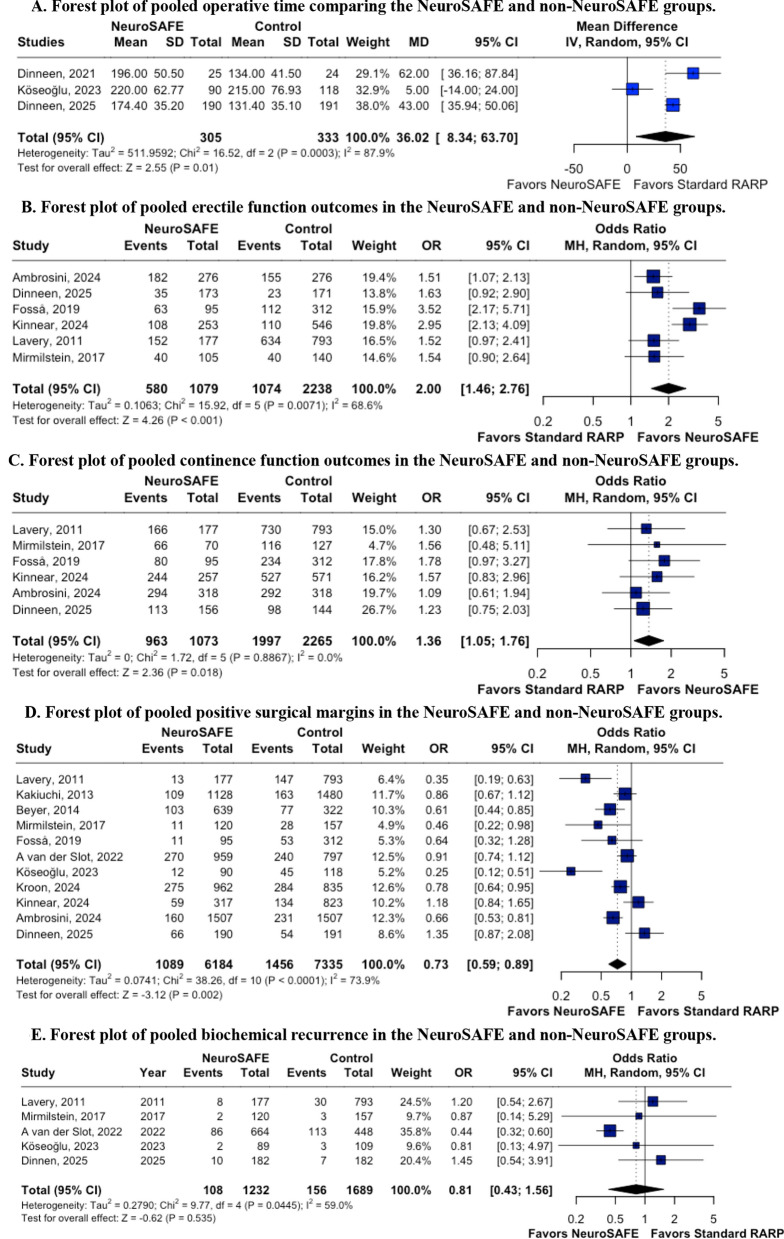
Forest plot analysis of operative time, erectile function, continence recovery, positive surgical margins, andbiochemical recurrence

Three studies [16, 17, 22] evaluated operative time in patients undergoing RARP with the NeuroSAFE technique, including 638 participants—305 in the NeuroSAFE group and 333 in the non-NeuroSAFE group (Fig. 3A). The pooled analysis demonstrated a significantly longer operative time in the NeuroSAFE group (mean difference: 36.02 minutes; 95% CI 8.34–63.70; *p* = 0.011). Substantial heterogeneity was observed (I^2^ = 87.9%). Assessment of publication bias using Egger’s test was not performed due to the limited number of studies.

### Erectile function

Six studies [16, 19, 21, 24, 25, 28] evaluated erectile function in patients who underwent RARP using the NeuroSAFE technique, including a total of 3,317 participants — 1079 in the NeuroSAFE group and 2,238 in the non-NeuroSAFE group (Fig. 3B). Pooled analysis demonstrated a statistically significant improvement in postoperative erectile function among patients treated with the NeuroSAFE approach (OR = 2.00; 95% CI, 1.46–2.74; p < 0.001). Heterogeneity was substantial (I^2^ = 68,6%), although no evidence of publication bias was detected based on Egger’s test (p = 0.64).

### Continence

Six studies [16, 19, 21, 24, 25, 28] assessed postoperative continence outcomes in patients undergoing RARP with the NeuroSAFE technique, encompassing 3,338 participants - 1,073 in the NeuroSAFE group and 2,265 in the non-NeuroSAFE group (Fig. 3C). The meta-analysis demonstrated a significantly higher rate of final urinary continence recovery among patients treated with the NeuroSAFE technique (OR = 1.36; 95% CI, 1.05–1.76; p = 0.018). Heterogeneity was minimal (I^2^ = 0%), and Egger’s test indicated no evidence of publication bias (p = 0.5).

### Positive surgical margins

Eleven studies [16, 18–25, 27, 28] evaluated positive surgical margin (PSM) rates in patients undergoing RARP with the NeuroSAFE technique, comprising a total of 13,519 participants—6184 in the NeuroSAFE group and 7335 in the non-NeuroSAFE group (Fig. 3D). The pooled analysis demonstrated a significantly lower likelihood of PSMs among patients treated with NeuroSAFE (OR = 0.73; 95% CI, 0.59–0.89; p = 0.001). Heterogeneity was considerable (I^2^ = 75.9%), while Egger’s test revealed no evidence of publication bias (p = 0.21).

### Biochemical recurrence

Five studies [16, 22, 24, 25, 27] evaluated biochemical recurrence (BCR) rates in patients undergoing RARP with the NeuroSAFE technique, including 2,921 participants—1,232 in the NeuroSAFE group and 1,689 in the non-NeuroSAFE group (Fig. 3E). The pooled analysis showed no statistically significant difference in BCR between groups (OR = 0.81; 95% CI, 0.43–1.56; p = 0.53). Moderate heterogeneity was observed (I^2^ = 59%), and Egger’s test indicated no evidence of publication bias (p = 0.13).

## Discussion

In this systematic review and meta-analysis of 13 studies evaluating patients who underwent RARP with the NeuroSAFE technique, we compared both functional and oncologic outcomes. Overall, 10,608 men underwent RARP with the NeuroSAFE technique, while 11,575 underwent non–NeuroSAFE RARP. Pooled analyses demonstrated statistically significant improvements in erectile function and urinary continence among patients treated with NeuroSAFE, along with a significant reduction in PSMs rates. No inferiority was observed in biochemical recurrence outcomes.

The findings of this review align with those of the recent randomized controlled trial [16], which reported lower incontinence rates and greater improvement in erectile function among patients treated with the NeuroSAFE technique. Importantly, no serious adverse events or deaths were attributed to the intervention.

The rationale behind the NeuroSAFE technique is that a more liberal nerve-sparing dissection—while theoretically increasing the risk of residual tumor—can be performed safely when paired with real-time intraoperative frozen section analysis. This strategy allows surgeons to maximize preservation of the neurovascular bundles and thereby improve postoperative erectile function, without compromising long-term oncologic control [21]. Despite its advantages, the NeuroSAFE technique has notable limitations, including the additional cost of pathology personnel, laboratory resources, and the extra operative time required—typically an additional 20–30 minutes per case [18]. Previous studies [17, 25] have estimated the incremental cost of implementing NeuroSAFE to range from £550 to £1000.

Younger age, lower oncologic risk, and the use of nerve-sparing techniques were independently associated with better postoperative sexual function, while age alone predicted continence recovery in the analysis by Haeuser et al. (2023) [29]. These results highlight the strong influence of baseline patient characteristics—particularly age—on functional outcomes after radical prostatectomy, while also emphasizing the benefit of maximizing nerve preservation when oncologically permissible.

Patients with high-risk features, such as Gleason 8–10, cT3–4 stage, PSA >20 ng/mL, or cN1 disease, consistently demonstrated poorer oncologic outcomes, reinforcing the need for careful selection when considering aggressive nerve-sparing [30]. In this context, Mirmilstein et al. [25] demonstrated higher postoperative potency rates with NeuroSAFE-assisted nerve sparing, supporting evidence that NeuroSAFE can safely expand bilateral nerve-sparing eligibility without compromising cancer control [22].

In the studies included in this systematic review, bilateral nerve sparing was performed in the majority of cases. For example, Koseoglu et al. (2023) [22] reported that bilateral nerve sparing was achieved in 81.1% of patients in the NeuroSAFE group compared with 55% in the standard RARP group (p < .05) [22]. Similarly, in the cohort studied by Kroon et al. (2024), the NeuroSAFE technique was applied bilaterally in 898 patients (93%) and unilaterally in 64 patients (7%) [23].

Analyzing surgical duration, the NeuroSAFE approach appears to be more time-consuming than the standard technique. However, during radical prostatectomy, additional steps—such as hemostasis and pelvic lymph node dissection, can be performed while awaiting the intraoperative assessment. Furthermore, emerging technologies, such as Histolog, may help shorten analysis time [31].

Across studies evaluating PSMs, patient selection and disease risk influenced outcomes. Mirmilstein et al. [25] limited RALP to high-risk patients staged ≤T3a, while Fossa et al. examined a more uniform cT2 cohort. In the largest analysis, Kroon et al. [23] reported a 29% PSM rate in a centralized NeuroSAFE cohort, with most margins measuring ≤3 mm. After adjustment for clinicopathologic factors, NeuroSAFE was independently associated with significantly lower odds of PSMs (OR 0.70; 95% CI 0.56–0.88), including fewer margins >1 mm and >3 mm.

Quality of life is closely linked to satisfaction with postoperative outcomes for both patients undergoing radical prostatectomy and their partners [32]. Although preoperative erectile dysfunction strongly influences postoperative potency, the randomized controlled trial [16] showed that the improvement in erectile function at 12 months (as measured by IIEF-5 scores) in the NeuroSAFE group was most likely driven by the higher rates of bilateral nerve-sparing achieved with this technique. This is consistent with the well-established relationship between nerve-sparing and recovery of erectile function.

This review has several strengths, including the incorporation of a large randomized controlled trial [16] and robust evidence across both functional and oncologic domains. Overall, the NeuroSAFE technique was associated with significant improvements in postoperative erectile function and urinary continence, as well as a markedly reduced risk of positive surgical margins. Importantly, NeuroSAFE did not demonstrate any increase in biochemical recurrence compared with standard RARP, supporting its safety and effectiveness in optimizing nerve-sparing without compromising oncologic control.

Our findings are consistent with those reported by Tingxuan Lv et al. [33], who also observed favorable functional and oncologic outcomes associated with the NeuroSAFE technique during robot-assisted radical prostatectomy. However, methodological differences should be considered. While their review included a broader range of observational studies with heterogeneous designs, the present meta-analysis applied stricter inclusion criteria, focusing exclusively on comparative studies directly evaluating NeuroSAFE-guided RARP versus standard RARP, allowing a more direct comparison between the two surgical approaches.

Another strength of our study is the consistently high diagnostic accuracy of the NeuroSAFE protocol when compared with final paraffin-embedded pathology. Across multiple studies, NeuroSAFE achieved sensitivities of 90% to 100% and specificities of 97% to 99%, with excellent concordance between intraoperative frozen sections and permanent margins [17, 23, 25, 26]. Although some authors have noted potential challenges in interpreting frozen sections [20], the overall evidence supports NeuroSAFE as a reliable tool for intraoperative margin assessment.

Our study has several limitations. First, selection bias across the included cohorts may have influenced both functional and oncologic outcomes. Several studies reported imbalances in baseline characteristics between NeuroSAFE and non-NeuroSAFE groups. For example, Schlomm et al. [26] noted worse tumor characteristics in the NeuroSAFE cohort, while Kakiuchi et al. [20] observed significant differences in age, biopsy Gleason score, and pathological stage. Mirmilstein et al. [25] found that patients undergoing NeuroSAFE were younger and had a higher proportion of high-grade and high-risk disease, whereas the non-NeuroSAFE group had more low-grade cancers. Similarly, Slot et al. [27] reported that NeuroSAFE patients were older and presented with higher clinical stage, Gleason grade, and D’Amico risk group, findings echoed by Koseoglu et al. [22] and Kroon et al. [23], who also demonstrated that NeuroSAFE cohorts contained patients with more adverse pathological features. These imbalances introduce the possibility that observed differences in outcomes may be influenced, at least in part, by underlying patient risk profiles rather than the intervention alone.

Second, heterogeneity in inclusion criteria across studies may further limit comparability. For instance, Dinneen et al. [16] included only patients with localized, operable prostate cancer and required good baseline erectile function (IIEF-5 > 21), a criterion not applied consistently across other cohorts. In addition, variability in the definitions and assessment tools used for functional outcomes (erectile function and continence) may further impact comparability; however, only studies with clearly defined and extractable outcomes were included in the quantitative synthesis to mitigate this limitation (Supp. Table 1).

Another limitation is that NeuroSAFE does not assess urethral or bladder neck margins across the entire prostate. Intraoperative frozen-section evaluation is limited to prostate margins adjacent to the neurovascular bundles, which may correlate with adverse pathological features on final radical prostatectomy specimens [34, 35]. 

Finally, findings from multivariable analyses underscore the importance of baseline characteristics as major determinants of outcomes regardless of NeuroSAFE use. Kinnear et al. [21] showed that biochemical recurrence was primarily associated with factors such as higher PSA, Gleason grade, nodal positivity, and positive margins rather than the surgical technique itself. Similarly, older age and preoperative erectile dysfunction were the strongest predictors of postoperative incontinence and impotence.

## Conclusions

This systematic review and meta-analysis demonstrates that, despite increased operative time, the NeuroSAFE approach was associated with improved early functional recovery and lower rates of positive surgical margins, without evidence of compromised oncological control as indicated by biochemical recurrence. However, given the predominance of nonrandomized studies and the observed heterogeneity, well-designed randomized trials and implementation studies are warranted to confirm these findings.

## Supplementary Information

Below is the link to the electronic supplementary material.

Supplementary file 1.

Supplementary file 2.

## Data Availability

No datasets were generated or analysed during the current study.

## References

[1] Siegel RL, Kratzer TB, Giaquinto AN, Sung H, Jemal A (2025) Cancer statistics, 2025. CA Cancer J Clin 75(1):10–45. 10.3322/caac.21871 39817679 10.3322/caac.21871 PMC11745215

[2] Kang SG et al (2020) ”Lessons learned from 12, 000 robotic radical prostatectomies: Is the journey as important as the outcome?”. Invest clinical Urolo. 61(1):1–10 10.4111/icu.2020.61.1.1 PMC6946819 31942457

[3] Yaxley JW, Coughlin GD, Chambers SK, Occhipinti S, Samaratunga H, Zajdlewicz L, Dunglison N, Carter R, Williams S, Payton DJ, Perry-Keene J, Lavin MF, Gardiner RA (2016) Robot-assisted laparoscopic prostatectomy versus open radical retropubic prostatectomy: early outcomes from a randomised controlled phase 3 study. Lancet. 10;388(10049):1057-1066. 10.1016/S0140-6736(16)30592-X. Epub 2016 Jul 26. Erratum in: Lancet. 2017 Apr 8;389(10077):e5. 10.1016/S0140-6736(17)30903-0. PMID: 27474375 10.1016/S0140-6736(16)30592-X 27474375

[4] Michl U et al (2016) Nerve-sparing surgery technique, not the preservation of the neurovascular bundles, leads to improved long-term continence rates after radical prostatectomy. Eur Urol 69:584 26277303 10.1016/j.eururo.2015.07.037

[5] Avulova S et al (2018) The effect of nerve sparing status on sexual and urinary function: 3-year results from the CEASAR study. J Urol 199:1202 29253578 10.1016/j.juro.2017.12.037

[6] Hoeh B, Preisser F, Zattoni F, Kretschmer A, Westhofen T, Olivier J, Soeterik TFW, van den Bergh RCN, Mandel P, Graefen M, Tilki D (2025) EAU-YAU prostate cancer working group. risk of biochemical recurrence and metastasis in prostate cancer patients treated with radical prostatectomy after a 10-year disease-free interval. Eur Urol Oncol 8(2):372–373. 10.1016/j.euo.2024.08.008 39306583 10.1016/j.euo.2024.08.008

[7] Schlomm T, Tennstedt P, Huxhold C, Steuber T, Salomon G, Michl U, Heinzer H, Hansen J, Budäus L, Steurer S, Wittmer C, Minner S, Haese A, Sauter G, Graefen M, Huland H (2012) Neurovascular structure-adjacent frozen-section examination (NeuroSAFE) increases nerve-sparing frequency and reduces positive surgical margins in open and robot-assisted laparoscopic radical prostatectomy: experience after 11,069 consecutive patients. Eur Urol 62(2):333–340. 10.1016/j.eururo.2012.04.057 22591631 10.1016/j.eururo.2012.04.057

[8] Page MJ, Moher D, Bossuyt PM, Boutron I, Hoffmann TC, Mulrow CD et al (2021) PRISMA 2020 explanation and elaboration: updated guidance and exemplars for reporting systematic reviews. BMJ 372:160. 10.1136/bmj.n160 10.1136/bmj.n160 PMC8005925 33781993

[9] Ketlyn Assunção Galhardo, Caio Vinicius Suartz. Frozen section analysis and NeuroSAFE in prostate cancer surgery: a systematic review and meta-analysis. PROSPERO 2025 CRD420251032774. Available from https://www.crd.york.ac.uk/PROSPERO/view/CRD420251032774.

[10] Ouzzani M, Hammady H, Fedorowicz Z, Elmagarmid A (2016) Rayyan — a web and mobile app for systematic reviews. Syst Rev 5:210. 10.1186/s13643-016-0384-4 27919275 10.1186/s13643-016-0384-4 PMC5139140

[11] R Core Team (2023). _R: A Language and Environment for Statistical Computing. R Foundation for Statistical Computing, Vienna, Austria. <https://www.R-project.org/>.

[12] Cochran WG (1950) The comparison of percentages in matched samples. Biometrika 37(3–4):256–266 14801052

[13] Higgins JPT, Thompson SG, Deeks JJ, Altman DG (2003) Measuring inconsistency in meta-analyses. BMJ 327(7414):557–560 12958120 10.1136/bmj.327.7414.557 PMC192859

[14] Sterne JA, Hernán MA, Reeves BC et al (2016) ROBINS-I: a tool for assessing risk of bias in non-randomised studies of interventions. BMJ 355:i4919 27733354 10.1136/bmj.i4919 PMC5062054

[15] Sterne JAC HM, McAleenan A, Reeves BC, Higgins JPT (2023) Cochrane handbook for systematic reviews of interventions version 6.4 (updated August 2023). Chapter 25: Assessing risk of bias in a non-randomized study. Cochrane

[16] Eoin D et al (2025) Effect of NeuroSAFE-guided RARP versus standard RARP on erectile function and urinary continence in patients with localised prostate cancer (NeuroSAFE PROOF): a multicentre, patient-blinded, randomised, controlled phase 3 trial. Lancet Oncol 26(4):447–458 40147459 10.1016/S1470-2045(25)00091-9

[17] Eoin D et al (2021) “NeuroSAFE frozen section during robot-assisted radical prostatectomy: peri-operative and histopathological outcomes from the NeuroSAFE PROOF feasibility randomized controlled trial.” BJU Intern 127(6):676–686 10.1111/bju.15256 32985121

[18] Burkhard B et al (2014) A feasible and time-efficient adaptation of NeuroSAFE for da Vinci robot-assisted radical prostatectomy. Eur Urol 66(1):138–144 24411279 10.1016/j.eururo.2013.12.014

[19] Fosså SD et al (2019) Improved patient-reported functional outcomes after nerve-sparing radical prostatectomy by using NeuroSAFE technique. Scand J Urol 53(6):385–391 31797716 10.1080/21681805.2019.1693625

[20] Kakiuchi Y et al (2013) Role of frozen section analysis of surgical margins during robot-assisted laparoscopic radical prostatectomy: a 2608-case experience. Hum Pathol 44(8):1556–1562 23561622 10.1016/j.humpath.2012.12.011

[21] Kinnear N et al (2024) Impact of frozen section on long‐term outcomes in robot‐assisted laparoscopic prostatectomy. BJU Intern 134(4):608–614 10.1111/bju.16437 38961710

[22] Köseoğlu E et al (2023) Intraoperative frozen section via neurosafe during robotic radical prostatectomy in the era of preoperative risk stratifications and primary staging with mpMRI and PSMA-PET CT: Is there a perfect candidate? Clin Genitourin Cancer 21(5):602–611 37451883 10.1016/j.clgc.2023.06.014

[23] Kroon LJ et al (2024) Centralized prostatectomy with intraoperative NeuroSAFE margin assessment improves surgical margin control. Histopathology 85(5):760–768 39108215 10.1111/his.15291

[24] Lavery HJ et al (2011) ‘Mohs surgery of the prostate’: the utility of in situ frozen section analysis during robotic prostatectomy. BJU Intern 107(6):975–979 10.1111/j.1464-410X.2010.09595.x 20880130

[25] Mirmilstein G et al (2018) The neurovascular structure‐adjacent frozen‐section examination (Neuro SAFE) approach to nerve sparing in robot‐assisted laparoscopic radical prostatectomy in a British setting–a prospective observational comparative study. BJU Intern 121(6):854–862 10.1111/bju.14078 29124889

[26] Schlomm T et al (2012) Neurovascular structure-adjacent frozen-section examination (NeuroSAFE) increases nerve-sparing frequency and reduces positive surgical margins in open and robot-assisted laparoscopic radical prostatectomy: experience after 11 069 consecutive patients. Eur Urol 62(2):333–340 22591631 10.1016/j.eururo.2012.04.057

[27] van Der Slot MA et al (2022) NeuroSAFE in radical prostatectomy increases the rate of nerve‐sparing surgery without affecting oncological outcome. BJU Intern 130(5):628–636 10.1111/bju.15771 PMC9796592 35536200

[28] Ambrosini F, Preisser F, Tilki D, Heinzer H, Salomon G, Michl U, Steuber T, Maurer T, Chun FKH, Budäus L, Pose RM, Terrone C, Schlomm T, Tennstedt P, Huland H, Graefen M, Haese A (2025) Nerve-sparing radical prostatectomy using the neurovascular structure-adjacent frozen-section examination (NeuroSAFE): results after 20 years of experience. Prostate Cancer Prostatic Dis 28(2):483–489. 10.1038/s41391-024-00851-x 38862777 10.1038/s41391-024-00851-x

[29] Haeuser L, Tully KH, Reicherz A, Berg S, Moritz R, Roghmann F, Noldus J, Palisaar RJ (2023) Functional outcome after radical prostatectomy in 1313 patients: a single-center study. Prostate 83(13):1290–1297. 10.1002/pros.24591 37350456 10.1002/pros.24591

[30] Ravi P, Xie W, Buyse M, Halabi S, Kantoff PW, Sartor O, Attard G, Clarke N, D’Amico A, Dignam J, James N, Fizazi K, Gillessen S, Parulekar W, Sandler H, Spratt DE, Sydes MR, Tombal B, Williams S, Sweeney CJ (2025) Refining risk stratification of high-risk and locoregional prostate cancer: a pooled analysis of randomized trials. Eur Urol 87(2):217–224. 10.1016/j.eururo.2024.04.038 38777647 10.1016/j.eururo.2024.04.038 PMC11579255

[31] Almeida-Magana R, Au M, Al-Hammouri T, Mathew M, Dinneen K, Mendes LST, Dinneen E, Vreuls W, Shaw G, Freeman A, Haider A (2025) Accuracy of the LaserSAFE technique for detecting positive surgical margins during robot-assisted radical prostatectomy: blind assessment and inter-rater agreement analysis. Histopathology 86(3):433–440. 10.1111/his.15336 39403832 10.1111/his.15336 PMC11707496

[32] Sanda MG, Dunn RL, Michalski J et al (2008) Quality of life and satisfaction with outcome among prostate-cancer survivors. N Engl J Med 358:1250–1261 18354103 10.1056/NEJMoa074311

[33] Lv T, Chen X, Zhang X, Yuan B, Zhang B, Wang L, Han X (2025) Neurovascular structure-adjacent frozen-section examination vs. standard robot-assisted radical prostatectomy: a systematic review and meta-analysis of two-arm comparative studies on functional and oncological outcomes. J Robo Surg 19(1):321. 10.1007/s11701-025-02486-z 10.1007/s11701-025-02486-z 40553352

[34] Billis A, Freitas LL, Magna LA, Samara AB, Ferreira U (2004) Prostate cancer with bladder neck involvement: pathologic findings with application of a new practical method for tumor extent evaluation and recurrence-free survival after radical prostatectomy. Int Urol Nephrol 36(3):363–368. 10.1007/s11255-004-0922-1 15783107 10.1007/s11255-004-0922-1

[35] Matalani CFA, Costa MSS, Rocha MRD, Lopes RI, Talizin TB, Bessa Júnior J, Nahas WC, Ribeiro-Filho LA, Suartz CV (2025) Minimally invasive radical prostatectomy versus open radical prostatectomy: a systematic review and meta-analysis of randomized control trials. Clinics (Sao Paulo, Brazil) 80:100636. 10.1016/j.clinsp.2025.100636 40294454 10.1016/j.clinsp.2025.100636 PMC12059318

