# Supplementary material for: NeuroSAFE-guided robot-assisted radical prostatectomy versus standard RARP: systematic review and meta-analysis of comparative studies
Source: J Robot Surg. 2026 May 13;20(1):496. doi: 10.1007/s11701-026-03390-w (PMC13171698; doi:10.1007/s11701-026-03390-w)
Supplement: Supplementary file 2 [file 11701_2026_3390_MOESM2_ESM.docx]

**Supplementary Material**

*Search strategy:*

- Medical Literature Analysis and Retrieval System Online (MEDLINE)
  - ("Prostate Cancer" OR "Prostatic Neoplasms" OR "Prostate Tumor") AND ("Nerve-Sparing" OR "Nerve Preservation" OR "Neurovascular Bundle Preservation") AND ("Robot-Assisted Radical Prostatectomy" OR "RARP") AND ("Frozen Section Analysis" OR "Neurovascular Structure-Adjacent Frozen-Section Examination" OR "NeuroSAFE")

**Supplementary Table 1.  Summary of Outcome Analysis Characteristics**

| Study (n = 13) | Erectile Function | Continence | Positive Surgical Margins | BCR Positive | Operative Duration | | |
| --- | --- | --- | --- | --- | --- | --- | --- |
|  |  |  |  |  | Overall | NS | Non-NS |
| Lavery, 2011 | | | | | 125 | 131 | 124 |
| *Follow up* | Baseline, 6 weeks, and every 3 months during the first postoperative year | |  |  |  |  |  |
| *Definition* | IPSS and SHIM scores.  Potency was defined as  a SHIM score ≥ 16 in patients who were  preoperatively potent (SHIM ≥ 16). | Use of either no pads or one security pad daily. | Tumour cells present at the inked margin. PSMs were dichotomized into ‘focal’ or ‘extensive’ if the length of the margin was < 2 mm or > 2 mm, respectively. | Post-prostatectomy PSA  > 0.2 ng/mL. |  |  |  |
| Schlomm, 2012 |  |  | At least one invasive malignant gland has contact with the inked SM | PSA level ≥ 0.2 ng/ml and rising  after RP. |  |  |  |
| Kakiuchi, 2013 |  |  | The presence of cancer cells at any area of the inked surface was considered a +SM.  Tumor presence when the tissue was shaved was also interpreted as a +SM at the bladder neck. |  |  |  |  |
| Beyer, 2014 |  |  |  |  |  |  |  |
| Mirmilstein, 2017 | | | | | | | |
| *Follow up* | ≥12 months | |  |  |  |  |  |
| *Definition* | Satisfactory erection for penetrative intercourse with or without a phosphodiesterase type 5 (PDE-5) inhibitor. | Used no pads or one precautionary pad. | A PSM was reported if on the ultimate histological analysis tumour was present at the inked margin outside the previously analysed/painted area of the frozen section (e.g. apex, base, posterior, anterior or through the NVB) or in the rare event of a false-negative frozen section. | PSA level of >0.2 ng/mL after RALP |  |  |  |
| Fossa, 2019 | Post-RP Sexual Summary Score (SexSS) .  ‘Erectile dysfunction’ was defined by patients’ dichotomized response to Item 10 of EPIC-26, indicating that ‘erection was never achieved or in less than half of the occasions when it was wanted’. | UrInSS of EPIC-26 and ‘Pad use’ was dichotomize (0 vs  1 pad). |  |  |  |  |  |
| Dinnen, 2021 |  |  |  |  |  |  |  |
| Dinnen, 2025 | | | | |  | 174.4 (SD = 35.2) | 131.4 (SD = 35.1) |
| *Follow up* | 3, 6, and 12 months after RARP | |  |  |  |  |  |
| *Definition* | IIEF-5 scores > 15 | ICIQ scores of < 5 | Positive surgical margin rates (surgical margin status was categorised as negative, small positive [unifocal and <3 mm], or significant positive [multifocal or ≥3 mm]) | Biochemical recurrence [PSA < 0.2 ng/mL followed by a rise above this level |  |  |  |
| Van der Slot, 2022 |  |  | Patients with a negative outer surface margin of the secondary resection after a PSM finding on IFS analysis were considered to have a final negative surgical margin at this site. | Biochemical recurrence (BCR) was defined as the first PSA level ≥0.2 ng/mL after RP. |  |  |  |
| Köseoglu, 2023 |  |  | - | Biochemical recurrence (BCR) was defined as a serum PSA level ≥0.2 ng/mL |  | 220 (Range: 90-400) | 215 (Range: 90-480) |
| Kroon, 2024 |  |  | At least one malignant tumour gland extending into the ink. |  |  |  |  |
| Kinnear, 2024 | | | | | | | |
| *Follow up* | ≥ 12 months |  |  |  |  |  |  |
| *Definition* | Scores 0–1 were considered to represent potency.  Erections adequate for  penetrative intercourse occurring pontaneously (0), with oral medications (1), intra-cavernosal injection (2) or inadequately (3). | Scores 0–1 were considered to represent continence.  pad free (0), safety pad but minimally wet (1), 2–3 pads/day (2) and ≥4 pads/day (3) | - | Two consecutive PSA values >0.2 ng/L |  |  |  |
| *Ambrosini, 2025* |  |  |  |  |  |  |  |
| *Follow up* | 12 months |  |  |  |  |  |  |
| *Definition* | Potency was defined as a score ≥2 on question 2 of the International Index of Erectile Function-5 (IIEF-5) | Continence was defined as the use of 0–1 safety pad per day | Tumour cells present at the inked margin |  |  |  |  |

Abreviations: IPSS = ; SHIM = Sexual Health Inventory for Men; NS = NEURO-Safe; SM = Surgical Margin; UrInSS = Urinary Incontinence SubScale. IIEF = International Index of Erectile Function, ICIQ = International Consultation on Incontinence Questionnaire Short Form

**Supplementary Figure 1.  Funnel Plot of Included Studies**


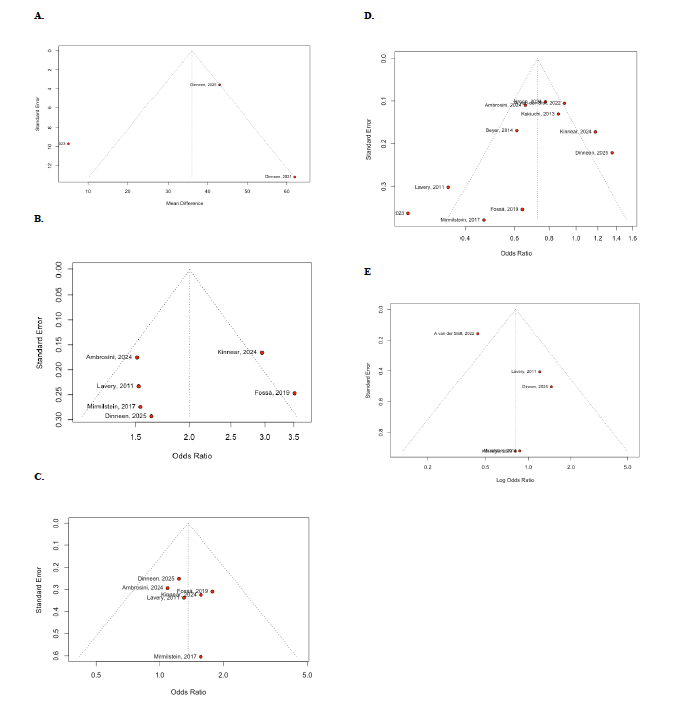


*Abreviations: A = Surgical Duration; B = Erectile Function; C = Continence; D = Positive Surgical Margin, E = Biochemical Recurrence*
